# Measurement of the adult human midbrain with transcranial ultrasound

**DOI:** 10.1371/journal.pone.0247920

**Published:** 2021-03-01

**Authors:** Karl Aoun, Kay L. Double, Verity Pearson-Dennett, Rezzak Yilmaz, Daniela Berg, Gabrielle Todd

**Affiliations:** 1 Brain and Mind Centre and Discipline of Pharmacology, Faculty of Medicine and Health, School of Medical Sciences, The University of Sydney, Sydney, NSW, Australia; 2 UniSA Clinical and Health Sciences and Alliance for Research in Exercise, Nutrition and Activity (ARENA), University of South Australia, Adelaide, SA, Australia; 3 Klinik für Neurologie, UKSH, Campus Kiel, Christian-Albrechts-Universität, Kiel, Germany; Institut de Biomedicina de València-CSIC, SPAIN

## Abstract

**Background:**

Transcranial sonography is increasingly used to aid clinical diagnoses of movement disorders, for example, to identify an enlarged area of substantia nigra echogenicity in patients with Parkinson’s disease.

**Objective:**

The current study investigated characteristics of the midbrain at the anatomical plane for quantification of substantia nigra echogenicity. METHODS: Area of substantia nigra echogenicity, cross-sectional area of the midbrain, and interpeduncular angle were quantified in two groups of adults aged 18–50 years: 47 healthy non-drug-using controls (control group) and 22 individuals with a history of methamphetamine use (methamphetamine group), a cohort with a high prevalence of enlarged substantia nigra echogenicity and thus risk of Parkinson’s disease.

**Results:**

In the control group, cross-sectional area of the midbrain (4.47±0.44 cm^2^) and interpeduncular angle were unaffected by age, sex, or image acquisition side. In the methamphetamine group, cross-sectional midbrain area (4.72±0.60 cm^2^) and area of substantia nigra echogenicity were enlarged compared to the control group, and the enlargement was sex-dependent (larger in males than females). Whole midbrain area and interpeduncular angle were found to be weak predictors of area of substantia nigra echogenicity after accounting for group and sex.

**Conclusions:**

History of methamphetamine use is associated with an enlarged midbrain and area of substantia nigra echogenicity, and the abnormality is more pronounced in males than females. Thus, males may be more susceptible to methamphetamine-induced changes to the brainstem, and risk of Parkinson’s disease, than females.

## Introduction

Transcranial sonography can be used to investigate the echogenic appearance of the substantia nigra (SN) [1], a midbrain structure that is challenging to visualize with other imaging modalities. The area of SN echogenicity is abnormally large in up to 90% of patients with Parkinson’s disease (PD) [2], with a high sensitivity (83–84%) and specificity (85–87%) for the disease [3, 4]. Approximately 9% of healthy adults also exhibit this abnormality [1], which is associated with increased iron [5], activation of microglia [6], decreased neuromelanin [7] and increased risk of PD [2]. Collectively, the data suggest that an enlarged area of SN echogenicity is a risk marker of PD.

Widespread adoption of this risk marker in clinical practice is limited by several factors, including, for example, operator dependency, differing quality of the pre-auricular acoustic bone window between patients [8], and differing image resolution between ultrasound machines and manufacturers such that normative data from one machine is not comparable to that of another. This is problematic given that the threshold value for abnormal area of SN echogenicity is based on normative data (e.g. 90^th^ percentile) [8, 9]. Little is known about the relationship between area of SN echogenicity and midbrain size. Hemi-midbrain area does not appear to vary with age in healthy adults [10, 11] and does not differ between PD patients and healthy controls [11]. Conversely, the SN:midbrain ratio significantly increases with age in healthy individuals, primarily due to a smaller ratio in children [10, 11]. This ratio is also significantly larger in PD patients compared to healthy controls [11], suggesting the enlarged area of SN echogenicity in PD is not due to an enlarged midbrain.

Image pre-processing and automated segmentation and detection algorithm techniques [12, 13] have been explored for potential to improve visualization and quantification of SN echomorphology but quantitative data remain reliant on subjective identification of the correct SN anatomical plane. The aim of the current study was to investigate the characteristics of the midbrain at the anatomical plane for quantification of SN echogenicity. The primary parameters of interest were cross-sectional area of the whole midbrain and interpeduncular angle to determine whether midbrain characteristics predict area of SN echogenicity. The research question was explored in a sample of young-to-middle aged healthy adults to avoid marked age-related changes in midbrain morphology [14]. Two groups of young-to-middle aged adults were studied. The first group consisted of individuals with no history of methamphetamine use and thus low prevalence (~9%) of enlarged area of SN echogenicity and PD risk [1, 15]. The second group consisted of individuals with a history of methamphetamine use and thus high prevalence (~56%) of enlarged area of SN echogenicity and PD risk [15]. We hypothesized that the interpeduncular angle and midbrain area would not differ between groups. Evidence that supports this hypothesis is a comparable hemi-midbrain area in PD patients and healthy controls [11]. We also hypothesized that age and sex would not predict interpeduncular angle and midbrain area, and that these measures would be reliable across raters.

## Materials and methods

The following healthy adults were recruited via advertisement: n = 47 with no history of use of illicit stimulant drugs (control group) and n = 22 with a history of methamphetamine use on ≥5 occasions (15) (methamphetamine group). The study was conducted in Adelaide, Australia after obtaining written informed consent from each participant. Inclusion criterion were aged 18–50 yrs (to avoid marked age-related changes in midbrain morphology [14]) with a pre-auricular acoustic bone window suitable for transcranial ultrasound. The study was approved by the University of South Australia Human Research Ethics Committee. Some data has been published previously in a different form [15].

### Participant screening

Each participant underwent screening tests prior to participation, including a brief medical history questionnaire, handedness questionnaire [16], depression questionnaire (Beck Depression Inventory-II) [17], and a neuropsychological assessment (Logical Memory I and II [18], Verbal Trails and Verbal Fluency [19, 20], and Digit Span [21]). Participants completed a urine drug test (PSCupA-6MBAU, US Diagnostics Inc., Huntsville, Alabama, USA) and a drug history questionnaire to document lifetime use of alcohol, tobacco, and illicit drugs [15].

General exclusion criteria were insufficient bone window for transcranial ultrasound (3 participants) and history of i) neurological illness/injury (control group) prior to onset of illicit drug use (methamphetamine group–due to the association between methamphetamine use and affective disorders; [22]), ii) use of antipsychotic medication (due to uncertainty about whether there is a casual relationship between use of antipsychotic medications and SN echogenicity), and iii) frequent use of illicit opioids (>3 times/year during period of illicit drug use) because opioid-induced respiratory depression can lead to brain hypoxia (for review see [23]). Participants were also excluded if they had consumed cannabis in the past 12 hours or returned a positive urine test for illicit stimulant drugs or benzodiazepines; these exclusion criteria ensured informed consent could be considered valid and that performance on the neuropsychological tests was not affected by acute drug use. As tetrahydrocannabinol can remain in the body for up to 80 days after last use [24], participants with a positive urine test for cannabis could participate if they had not used cannabis in the past 12 hours.

### Transcranial sonography

**Fig 1:**
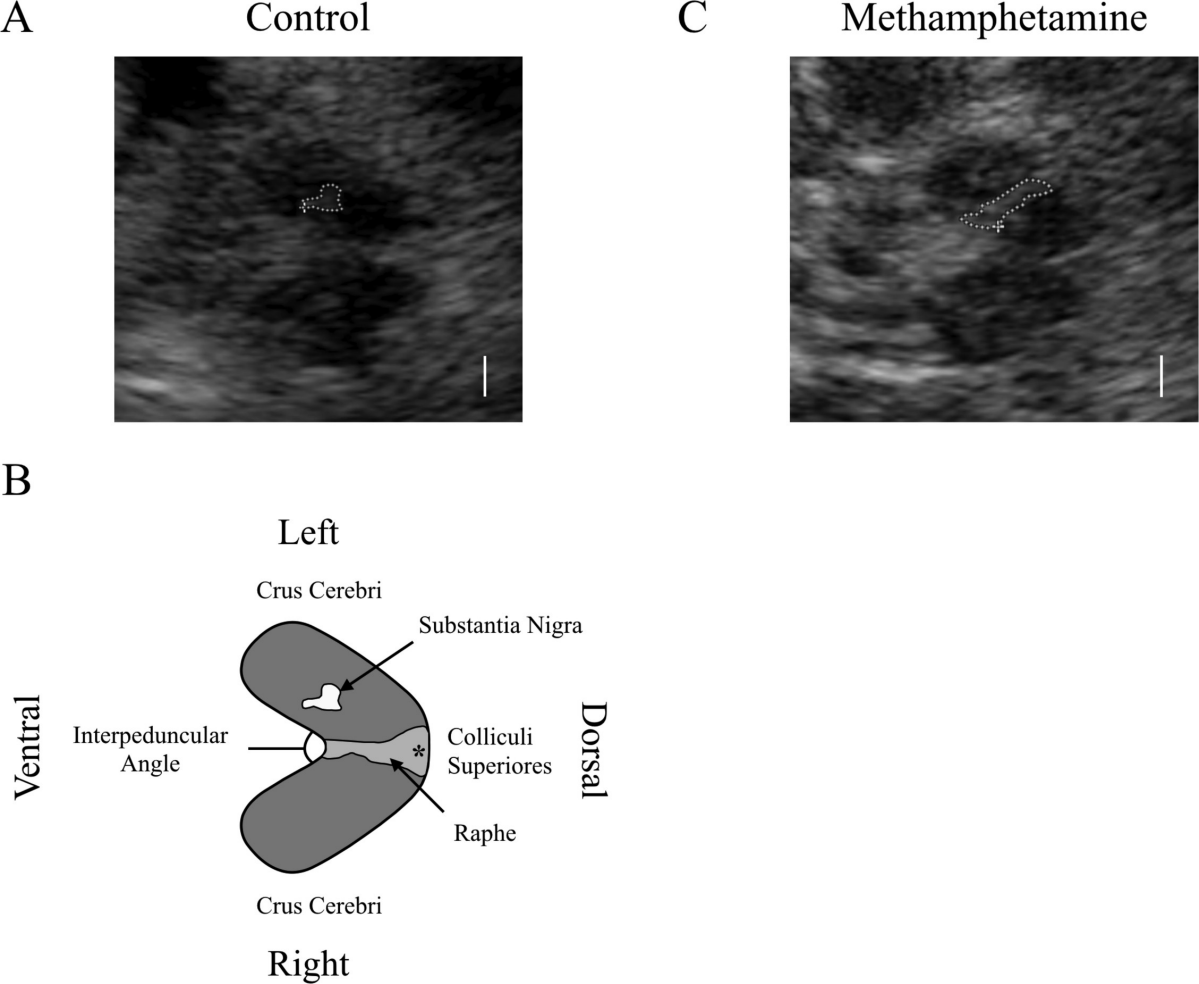
Raw sonographic images and a schematic drawing showing the midbrain (mesencephalic brainstem) in two participants. A) Control participant. The substantia nigra ipsilateral to the probe (the side at which the planimetric measurement is made) is encircled with a dotted line. B) Schematic drawing of image A showing anatomical landmarks and measurement parameters. The area shaded in dark gray represents the cross-sectional area of the midbrain. C) Methamphetamine participant. * Cerebral aqueduct. Raphe, echogenicity of midline structures.

Transcranial ultrasound of the SN was performed according to international guidelines [9] using a Philips iU22 ultrasound system (manufactured June 2004, refurbished November 2011 with software level 6.0.2.144) and 1–5 MHz transducer (s5-1, Philips Healthcare, Best, The Netherlands). The transducer was positioned over the pre-auricular acoustic bone window. Images were acquired (B-mode setting, penetration depth: 14–16 cm, dynamic range: 60 dB) from the right and left side by one experienced operator (GT) who has performed >400 examinations in healthy adults and patients [15]. A qualitative rating of the bone window was made (1: excellent, 2: good, 3: poor, 4: very poor) and area of SN echogenicity was measured at its greatest extent (ipsilateral to the transducer). Cross-sectional area of the whole midbrain (including cerebral aqueduct), hemi-midbrain area ipsilateral to the target SN, and angulation of the ventral surface of the midbrain in the fossa interpeduncularis (i.e. interpeduncular angle) were also measured on this image (Fig 1B).

### Measurement validity and reliability

Professor Daniela Berg (>25 years’ experience) and Dr Rezzak Yilmaz (5 years’ experience) performed a blinded qualitative assessment of image quality, measurement plane, and SN encirclement on 20 randomly-selected images acquired by the experienced operator (GT). The experienced operator (GT) measured all of the ultrasound parameters on all participants using in-built Philips iU22 software. Two additional raters then independently measured midbrain characteristics on all control participants in a blinded manner, for subsequent inter-rater reliability assessments. One of the additional raters (KD) is an experienced neuroanatomist (>20 years of experience examining post-mortem human midbrains) and is trained in SN sonography but has only performed the technique on a small number of participants (called ‘neuroanatomist’ rater). The other additional rater (KA) is minimally experienced in neuroanatomy with no formal training or experience in SN sonography (called ‘inexperienced’ rater). The neuroanatomist and inexperienced raters measured midbrain characteristics using free open-source DICOM viewing software (HOROS Project, version 3.0). Reproducibility of the two planimetric software packages (Philips iU22 versus HOROS Project) was tested by measuring the area of ten test shapes in each software package and comparing the two sets of measurements with intraclass correlation analysis. The intraclass correlation coefficient (ICC) for the test shapes was 1.0, indicating high reliability of area measurements obtained from the two software packages.

### Data analysis

Group data are presented as mean±standard deviation. All data were assessed for normality and equivalence of variance with Shapiro-Wilks test and Levene’s test, respectively. Between-group comparison of participant characteristics were made with unpaired Student’s t-test or Mann-Whitney U test (IBM SPSS Statistics 22, Armonk, USA). Statistical significance was set at P<0.05.

The effect of group, sex, age, lifetime use of alcohol and tobacco, and side (right, left) were investigated on the following outcome measures (measured by the experienced TCS rater, GT): whole midbrain area, interpeduncular angle, and area of SN echogenicity. The analysis involved mixed effects models with the following fixed effects: ‘Group’, ‘Sex’, ‘Age’, ‘Alcohol’, ‘Tobacco’, and ‘Side’ (Stata v.15.1, StataCorp, Texas, USA). A random-effects (participant ID) intercept was included to account for between-participant variability. Raw data that violated model assumptions (distribution and homoscedasticity of the residuals) were transformed and the models were run on the transformed data. Age and alcohol and tobacco use were not predictors of midbrain area or area of SN echogenicity and were therefore excluded from the final models. Group and sex were significant predictors of midbrain area and area of SN echogenicity, and thus were included in the final analysis. Contrasts were used to calculate the main effects of ‘Group’, ‘Sex’, ‘Side’, and ‘Group’-by-‘Sex’ interaction, and are reported as Chi square. Post-hoc comparisons in mixed effects models were performed (Bonferroni correction) to explore significant interactions. The effect of midbrain area and inter-peduncular angle on area of SN echogenicity were investigated with separate mixed effects models–and then adjusted to account for the effects of ‘Group’ and ‘Sex’. Model results are reported in text and tables as coefficients and 95% confidence intervals. A two-way ANOVA was used to investigate the effect of ‘Group’ (between-subject factor) and ‘Side’ (left, right; within-subject factor) on the SN echogenicity area:hemi-midbrain area ratio (IBM SPSS Statistics 22, Armonk, USA).

Inter-rater reliability between the three raters was assessed with Cronbach’s α and inter-rater correlation matrix (two data points per participant: right and left side). Reliability between the experienced TCS and neuroanatomist raters was assessed by intraclass correlation coefficient (ICC) determination using a two-way mixed effects model with absolute agreement (IBM SPSS Statistics 22, Armonk, USA). ICC values were qualitatively graded as: <0.5 = poor reliability, 0.5–0.75 = moderate reliability, 0.75–0.90 = good reliability, and >0.90 = excellent reliability [25].

## Results

### Participant characteristics

Table 1 shows participant characteristics for each group. The groups differed in age (P<0.001), weight (P = 0.003), years of education (P = 0.020), and performance on the Logical Memory II test (% recall: P = 0.012, % retention: P = 0.030), but not on other measures of neuropsychological performance. Nine participants in the methamphetamine group had been diagnosed with depression and/or anxiety after the onset of illicit drug use, but the groups did not differ in recent symptoms of depression (BDI-II score).

**Table 1:** Participant characteristics in the control and methamphetamine groups.

Parameter	Control group	Methamphetamine group
Sex	27 F, 20 M	11 F, 11M
Age (years)	23.8±7.1	34.2±7.4 *
Height (cm)	170±11	174±9
Weight (kg)	65±12	77±14 *
Handedness	39 R, 8 L	22 R, 0 L
Education (years)	16.1±2.3	14.5±3.4 *
BDI-II score	7.3±7.5	8.1±6.2
Mental disorder diagnosis	0	9
LM I (% recall)	55.1±14.4	48.2±13.5
LM II (% recall)	48.4±14.6	37.4±16.2 *
LM II (% retention)	87.5±13.5	76.0±18.3 *
FAS	39.4±8.8	44.1±13.4
Digit span forwards	6.9±1.0	7.1±1.3
Digit span backwards	5.3±1.2	5.2±1.5
13M (s)	32.6±16.8	36.9±19.6
Alcohol (drinks)	94% (858±2,104)	100% (13,976±16,709) *
Tobacco (cigarettes)	36% (15±43)	100% (93,713±115,072) *
Cannabis	13% (1±1)	100% (3,260±3,662)
Illicit stimulants		
Ecstasy	0%	96% (161±248)
Methamphetamine	0%	100% (811±1,208)
Cocaine	0%	82% (9±12)
Pharma stimulants	0%	36% (6±9)
Hallucinogens	0%	86% (92±124)
Inhalants	0%	55% (95±184)
Sedatives	2% (1)	55% (36±76)
Opioids	0%	59% (6±8)

Data presented are mean±standard deviation, except for lifetime drug use parameters which are presented as the percentage of participants who have used a class of drug and the mean±standard deviation for the lifetime occasions of use in brackets, in individuals who reported use of this class of drug. ‘Mental disorder diagnosis’ refers to the number of participants who had received a formal diagnosis of depression and/or anxiety after commencement of illicit drug use. The term ‘ecstasy’ includes MDMA (3,4-methylenedioxymethamphetamine) and MDA (3,4-methylenedioxyamphetamine). The term ‘pharma stimulants’ includes illicit use of methylphenidate and dexamphetamine. The term ‘hallucinogens’ includes LSD (lysergic acid diethylamide), ‘magic mushrooms’, DMT (dimethyltryptamine), 2CI/2CB (2,5-dimethoxy-4-iodophenethylamine or 2-(4-bromo-2,5-dimethoxyphenyl) ethanamine), ketamine, salvia divinorum, and datura. The term ‘inhalants’ includes amyl nitrate and nitrous oxide. The term ‘sedatives’ includes GHB/fantasy, methaqualone, and illicit use of benzodiazepines and pregabalin. The term ‘opioids’ describes opium, heroin, poppy tea, and illicit use of oxycodone, buprenorphine, morphine, and/or hydrocodeine. LM, Logical memory. * Significantly different from the control group (P<0.05).

Table 1 also shows group data for lifetime use of licit and illicit drugs. Estimated lifetime consumption of alcohol and tobacco was significantly higher in the methamphetamine group than in the control group (P<0.001). Illicit drug use was minimal in the control group and poly-drug use was common in the methamphetamine group. Most participants in the methamphetamine group reported a history of use of cannabis, ecstasy, cocaine, and/or hallucinogens, in addition to methamphetamine. Six participants in the methamphetamine group returned a positive urine test for cannabis and one participant in the control group returned a positive urine test for an opioid resulting from short-term use of a prescribed opioid for pain relief after a dental procedure.

### Transcranial ultrasound

The bone window qualitative rating on the right and left side was 1.1±0.4 and 1.2±0.5 for the control group and 1.5±0.8 and 1.4±0.6 for the methamphetamine group. The diameter of the third ventricle (largest side) was normal (<7 mm) for all participants and did not differ between groups (control: 1.7±0.8 mm, methamphetamine: 1.8±0.9 mm).

**Fig 2:**
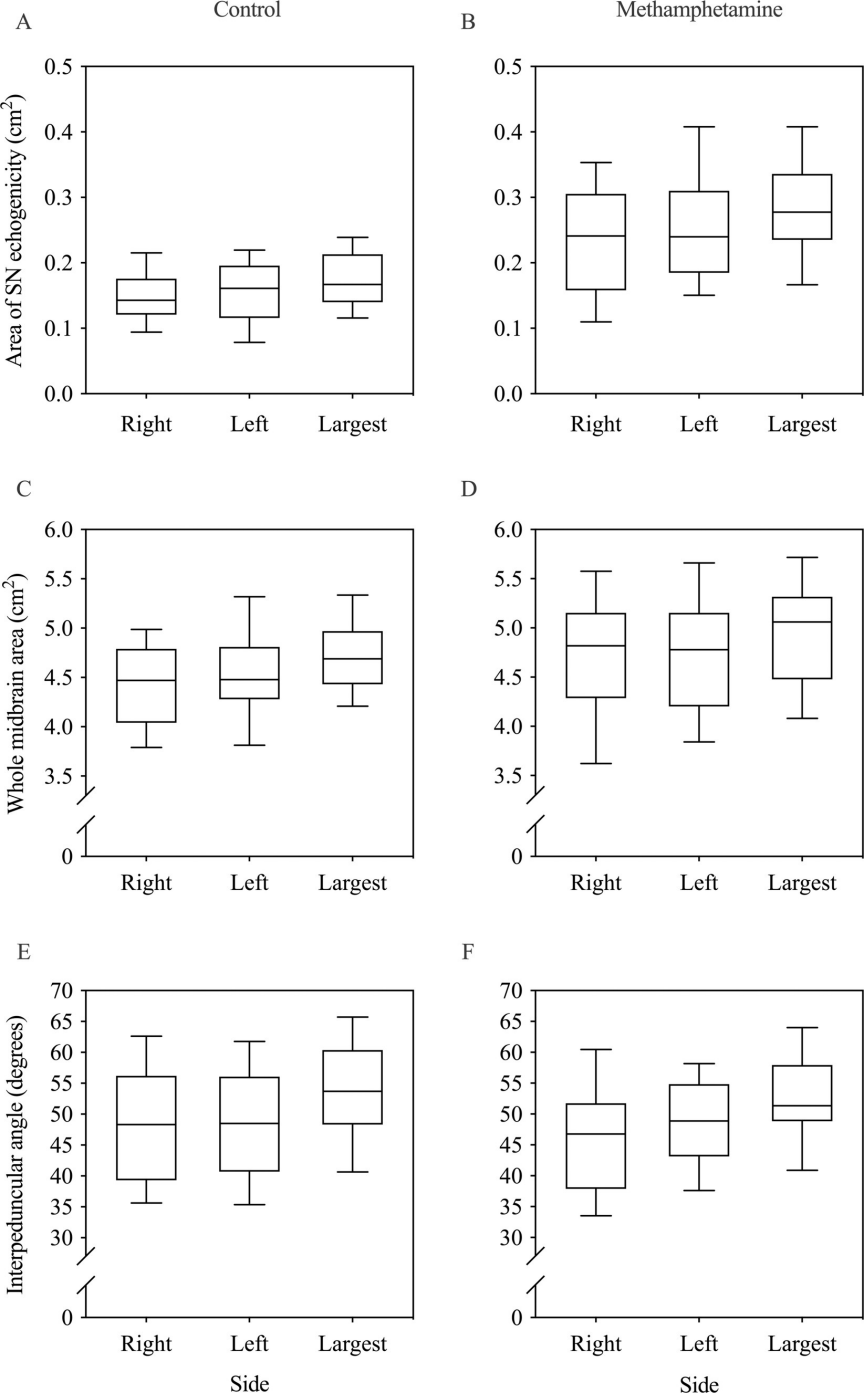
Group data showing the midbrain and substantia nigra measurements on the right and left side and largest side. The boundary of each box represents the 25^th^ and 75^th^ percentile and the whiskers (error bars) represent the 10^th^ and 90^th^ percentiles. The solid line within each box is the median. A and B) Area of substantia nigra (SN) echogenicity in the control group (A) and methamphetamine group (B). C and D) Cross-sectional area of the whole midbrain in the control group (C) and methamphetamine group (D). E and F) Interpeduncular angle in the control group (E) and methamphetamine group (F).

Mixed effects models were used to investigate the effect of group, sex, age, lifetime use of alcohol and tobacco, and side (right, left) on transcranial ultrasound outcome measures (measured by the experienced TCS rater). Fig 2A and 2B show group data for area of SN echogenicity. There was no significant main effect of age, side (right, left), or lifetime use of alcohol or tobacco on area of SN echogenicity, but there was a significant main effect of group (χ^2^(1) = 51.48, P<0.001) and sex (χ^2^(1) = 10.89, P = 0.001; Table 2). Area of SN echogenicity was significantly larger in the methamphetamine group than in the control group (average across sides: 0.244±0.076 versus 0.155±0.042 cm^2^) and significantly larger in males than females (average across sides and groups: 0.200±0.083 versus 0.169±0.052 cm^2^). There was also a significant group-by-sex interaction on area of SN echogenicity (χ^2^(1) = 13.01, P<0.001; Table 2). Males and females did not differ in the control group, but males had a significantly larger area of SN echogenicity than females in the methamphetamine group (average across sides: 0.288±0.072 versus 0.201±0.053 cm^2^; P<0.001). Furthermore, there was a significant difference in area of SN echogenicity between control and methamphetamine males (P<0.001) but there was only a trend for a difference between control and methamphetamine females (P = 0.058).

**Table 2:** Results of the mixed effects models showing the main effects of, and interactions between, group, sex, and side for whole midbrain area and area of substantia nigra echogenicity.

	β	95% CI	Sig
**Whole midbrain area**			
Intercept	4.562	4.364, 4.760	<0.001
Group	0.503	0.189, 0.817	0.002
Sex	-0.232	-0.478, 0.015	0.065
Side	0.078	-0.056, 0.211	0.254
Group x Sex	-0.536	-0.970, -0.103	0.015
**Substantia nigra area**			
Intercept	0.148	0.125, 0.170	<0.001
Group	0.135	0.100, 0.171	<0.001
Sex	0.004	-0.024, 0.032	0.788
Side	0.009	-0.006, 0.024	0.235
Group x Sex	-0.091	-0.140, -0.041	<0.001

Fig 2C and 2D show group data for cross-sectional area of the whole midbrain. There was no significant main effect of age, side (right, left), or lifetime use of alcohol or tobacco on whole midbrain area, but there was a significant main effect of group (χ^2^(1) = 4.51, P = 0.034) and sex (χ^2^(1) = 20.46, P<0.001; Table 2). Midbrain area was significantly larger in the methamphetamine group than in the control group (average across sides: 4.72±0.60 versus 4.47±0.44 cm^2^) and significantly larger in males than females (average across groups: 4.78±0.48 versus 4.36±0.45 cm^2^). There was a significant group-by-sex interaction on midbrain area (χ^2^(1) = 5.88, P = 0.015; Table 2). Males and females did not differ in the control group (P = 0.392), but males had a significantly larger midbrain area than females in the methamphetamine group (average across sides: 5.10±0.44 versus 4.34±0.48 cm^2^; P<0.001). Furthermore, there was a significant difference between control and methamphetamine males (P = 0.010) but not between control and methamphetamine females (P = 1.000). The average hemi-midbrain area across sides was 2.21±0.28 cm^2^ and 2.31±0.35 cm^2^ in the control and methamphetamine groups, respectively.

Fig 2E and 2F show group data for the interpeduncular angle. There was no significant main effect of group, sex, age, side (right, left), or lifetime use of alcohol or tobacco on interpeduncular angle and no significant interactions were observed.

**Fig 3:**
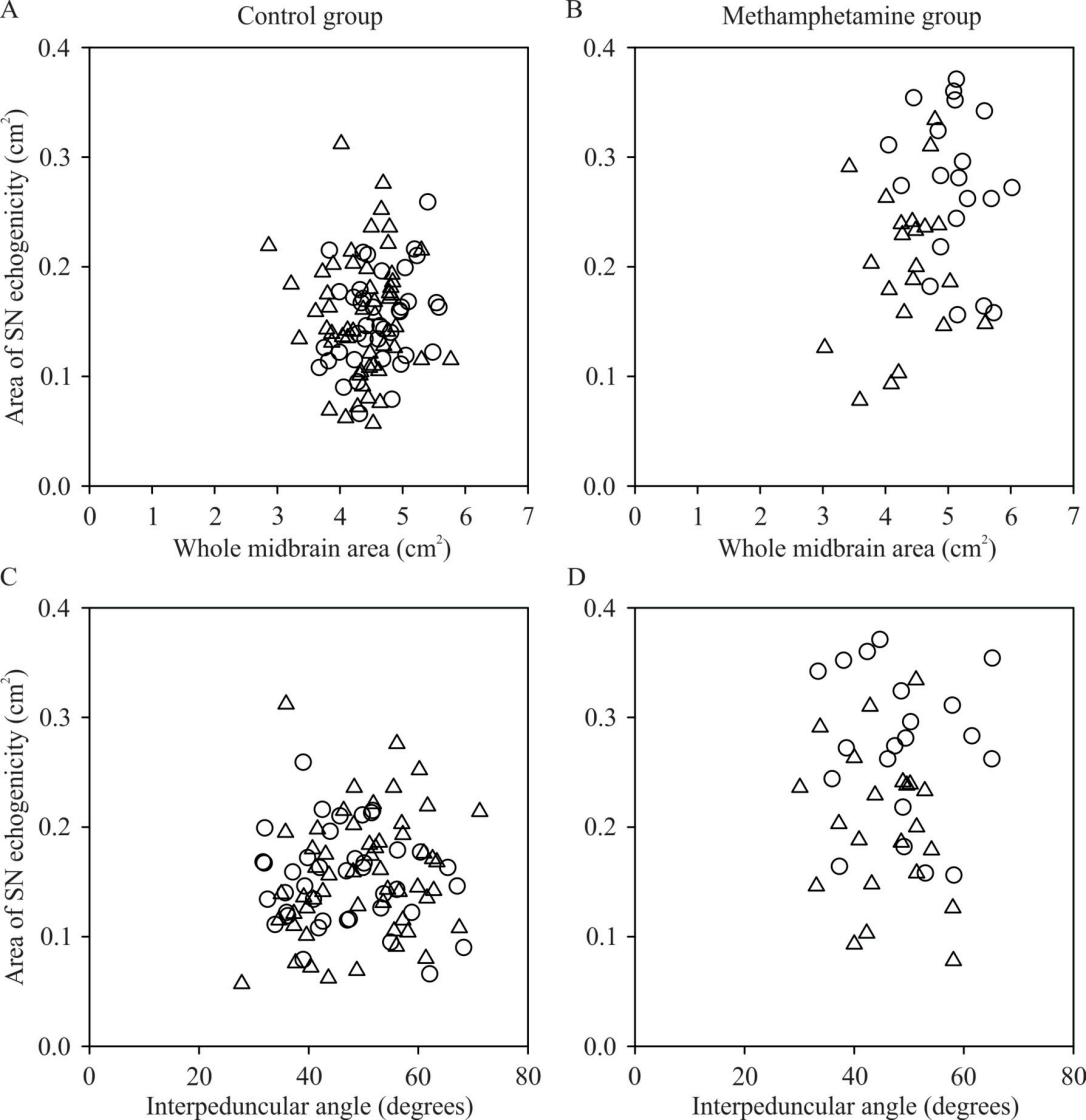
Individual data showing the relationship between midbrain characteristics (whole midbrain area and interpeduncular angle) and the area of substantia nigra (SN) echogenicity. A and B) Relationship between the whole midbrain area and area of SN echogenicity in the control group (A) and methamphetamine group (B). C and D) Relationship between interpeduncular angle and area of SN echogenicity in the control group (C) and methamphetamine group (D). Circles, males. Triangles, females.

The effect of i) whole midbrain area and ii) interpeduncular angle on the area of SN echogenicity was also investigated with separate mixed effects models. In the unadjusted model, midbrain area significantly predicted the area of SN echogenicity (P = 0.006; Table 3). A larger midbrain area was associated with a larger area of SN echogenicity. In the adjusted model, which accounted for group and sex, there was only a trend for midbrain area to predict area of SN echogenicity (P = 0.087; Table 3; Fig 3A and 3B). The interpeduncular angle also tended to predict area of SN echogenicity, but this association did not reach statistical significance in the unadjusted (P = 0.086) or adjusted (for group and sex; P = 0.077) models (Table 3; Fig 3C and 3D). The between-group difference in area of SN echogenicity was still evident when the area of SN echogenicity was expressed as a ratio of hemi-midbrain area (average across sides: control = 0.071±0.025, methamphetamine = 0.107±0.037; F_1,67_ = 29.697, P<0.001).

**Table 3:** Results of the mixed effects models showing the main effects of whole midbrain area, interpeduncular angle, side, group, and sex on the log transformed area of substantia nigra echogenicity.

	Unadjusted model			Adjusted model		
	β	95% CI	Sig	β	95% CI	Sig
**Log substantia nigra area**						
Intercept	-2.523	-3.047, -1.999	<0.001	-2.308	-2.889, -1.727	<0.001
Whole midbrain area	0.161	0.047, 0.275	0.006	0.097	-0.014, 0.208	0.087
Side	0.021	-0.069, 0.110	0.649	0.026	-0.064, 0.115	0.573
Group				0.419	0.236, 0.601	<0.001
Sex				-0.087	-0.237, 0.063	0.256
**Log substantia nigra area**						
Intercept	-2.048	-2.350, -1.746	<0.001	-2.110	-2.406, -1.813	<0.001
Interpeduncular angle	0.005	-0.001, 0.011	0.086	0.005	-0.001, 0.011	0.077
Side	0.029	-0.058, 0.117	0.510	0.029	-0.058, 0.117	0.509
Group				0.440	0.288, 0.593	<0.001
Sex				-0.130	-0.273, 0.012	0.073

### Inter-rater reliability

The inter-rater correlation matrix revealed an inconsistency between raters (Cronbach’s α: midbrain area = 0.803, interpeduncular angle = 0.818). There was a strong linear relationship (r>0.816) between the values obtained by the experienced TCS rater and neuroanatomist rater, however the linear relationship between these two raters and the inexperienced rater was weak (r<0.508). The ICC for the experienced and neuroanatomist raters revealed good to excellent reliability for single and average measures of midbrain area (ICC = 0.794–0.885) and interpeduncular angle (ICC = 0.974–0.987; Table 4).

**Table 4:** Inter-rater reliability for whole midbrain area and interpeduncular angle.

	Mean±SD	Experienced TCS rater	Neuro-anatomist rater	Inexperienced rater
**Whole midbrain area**				
Experienced TCS rater	4.47±0.53	1.000	0.816	0.508
Neuroanatomist rater	4.40±0.66	0.816	1.000	0.425
Inexperienced rater	4.33±0.55	0.508	0.425	1.000
Experienced TCS vs neuroanatomist	Cronbach’s α = 0.887, F = 8.842, P<0.001			
Single measures	ICC = 0.794, 95% CI = 0.705–0.858			
Average measures	ICC = 0.885, 95% CI = 0.827–0.924			
**Interpeduncular angle**				
Experienced TCS rater	48.43±10.10	1.000	0.973	0.435
Neuroanatomist rater	48.30±10.10	0.973	1.000	0.389
Inexperienced rater	47.24±10.12	0.435	0.389	1.000
Experienced TCS vs neuroanatomist	Cronbach’s α = 0.986, F = 74.034, P<0.001			
Single measures	ICC = 0.974, 95% CI = 0.960–0.982			
Average measures	ICC = 0.987, 95% CI = 0.980–0.991			

Data are from the control group with two data points per participant (right and left side). The mean±standard deviation (SD) is reported for each rater and the inter-rater correlation matrix is shown. Intraclass correlation (ICC) estimates (absolute-agreement, two-way mixed effects model) for the experienced TCS and neuroanatomist raters, and their 95% confidence intervals (CI), are also reported.

## Discussion

We show, for the first time, that area of SN echogenicity and cross-sectional area of the midbrain are significantly larger in young-to-middle aged adults with a history of methamphetamine use than in non-methamphetamine-using controls, and that this change is influenced by sex. The results also suggest that midbrain area and interpeduncular angle are weak predictors of area of SN echogenicity (at the correct image plane) after accounting for group and sex.

The results of the mixed effects models show that area of SN echogenicity and midbrain area do not vary with age, sex, side of image acquisition, or lifetime use of alcohol or tobacco in the control group. This finding supports the hypothesis. However, in the methamphetamine group, a population with a high prevalence of abnormal SN echogenicity [15], there was an effect of sex on area of SN echogenicity and midbrain area. Male methamphetamine users had a significantly larger midbrain and area of SN echogenicity than female methamphetamine users. This leads one to question whether changes in the SN lead to enlargement of the midbrain or vice versa. The latter is unlikely given that midbrain area only weakly predicted area of SN echogenicity after accounting for group and sex (P = 0.087). Furthermore, area of SN echogenicity is larger in PD patients [11] and methamphetamine users (current study) than in healthy controls when expressed as a ratio of hemi-midbrain area.

The significant group-by-sex interaction on area of SN echogenicity extends understanding of the relationship between methamphetamine use, SN echomorphology, and risk of PD [15]. Current understanding of this relationship includes the well-documented association between SN echogenicity and risk of Parkinson’s disease in older adults (e.g. [26]) and the observation that adults with a history of methamphetamine use exhibit abnormal SN echogenicity and clinical signs of parkinsonism [15]. Analysis of hospital records also shows that the prevalence of Parkinson’s disease is significantly higher in adults that have previously entered the health system for a methamphetamine-related problem [27]. The results of the current study suggest that males may be more vulnerable to methamphetamine-induced changes in the SN, and thus risk of Parkinson’s disease, than females, an effect overlooked in the original 2016 study [15] because the groups were matched for sex. A sex-effect of methamphetamine is also evident in the motor cortex, with male methamphetamine users exhibiting greater changes in motor cortical excitability than female methamphetamine users [28]. This apparent vulnerability of males to methamphetamine-induced changes in motor circuitry could be related to levels of gonadal steroid hormones. Testosterone is toxic to dopaminergic neurons undergoing oxidative stress [29] and oxidative stress is a hallmark feature of methamphetamine toxicity [30]. Furthermore, estrogen has been shown to protect nigrostriatal dopaminergic neurons against neurotoxicity induced by MPTP or methamphetamine [31].

Age, sex, side, and lifetime use of alcohol and tobacco did not affect interpeduncular angle and interpeduncular angle was only a weak predictor of area of SN echogenicity (P = 0.077). The result supports the hypothesis and implies that small variations in interpeduncular angle do not significantly affect area of SN echogenicity at the optimal image plane for quantification of SN echomorphology. Normative data for interpeduncular angle, obtained from a larger sample of healthy adults, could thus conceivably be used to guide or confirm SN image plane selection–with a more acute or obtuse angle reflecting an abnormally low or high image plane, respectively.

Midbrain morphometric parameters require good-to-excellent reliability to be used as a guide for, or confirmation of, SN image plane selection. Good-to-excellent reliability was achieved between the experienced and neuroanatomist raters for interpeduncular angle (ICC = 0.974–0.987) and midbrain area (ICC = 0.794–0.885). Thus, experience with either TCS or neuroanatomy is sufficient to achieve reliable measures of interpeduncular angle and midbrain area. The hemi-midbrain areas reported in the current study are consistent with a previous study involving healthy adults aged 18–72 years (right side: 2.35±0.32 cm^2^; left side: 2.30±0.32 cm^2^) [10]. The weak correlation between the inexperienced rater and the experienced and neuroanatomist raters suggests that training would still play a key role in measurement and/or application of brainstem morphometric parameters in research and clinical settings.

A study limitation is the sample age-range (18–50 years). Further research is required to investigate brainstem echomorphic characteristics in older adults, and how these parameters, and their relationship to one another, change over time. Healthy adult females have a higher density of dopamine reuptake transporters in the striatum than males [32]. Thus, aging-related changes in dopaminergic neurons [33] could conceivably have a greater impact on males than females. Measurement of head or whole brain size may also have aided interpretation of the data.

In summary, history of methamphetamine use is associated with a sex-specific increase in area of SN echogenicity and midbrain area. The weak predictive effect of midbrain area and interpeduncular angle on area of SN echogenicity, suggest that normative midbrain area and angle data (derived from a larger data set) could be used to aid or confirm identification of the correct image plane for measurement of SN echogenicity.
